# A Medico-Legal Treatise on Malpractice and Medical Evidence

**Published:** 1860-09

**Authors:** 


ART. VIII.—*A Medico-Legal Treatise on Malpractice and Medical Evidence, comprising the Elements of Medical Jurisprudence.* By JOHN J. ELWELL, M.D., Member of the Cleveland Bar. 8vo., pp. 588. New York, 1860.

THE motto of this book, borrowed from an eminent criminal jurist, Mr. David Paul Brown, of the Philadelphia bar, should sink deeply into the heart and conscience of every physician and lawyer. "A doctor who knows nothing of law, and a lawyer who knows nothing of medicine, are deficient in the essential requisites of their respective professions." The importance of this twofold knowledge is everywhere insisted upon in the able work of Mr. Elwell, who, from his peculiar position as a physician, and a member of the bar, was admirably qualified for the task of supplying the two professions with a medico-legal treatise upon the subject before us. The following extract from his preface will serve to explain the motives which prompted him to undertake the work:—

"The active practice of medicine and surgery for several years having taught me something as to the magnitude of the duties and difficulties, the wants and liabilities, of the medical profession; and a corresponding length of time devoted to the study and practice of law having deeply impressed me with the importance of the two professions, relatively as well as independently considered, developing also the obvious fact that legal men and legal works devote too little attention to medico-legal subjects; I have thought that in no way could I better serve the interests of the two great professions to which I have devoted my life, and promote the great ends of science and justice, than by endeavoring to embody, in a concise, complete, and comprehensive work, all the settled principles and known authorities, as well as the result of my own thought and experience, upon the subject of malpractice and medical evidence."

The work of Dr. Elwell is, so far as our knowledge enables us to infer, the first that has ever appeared upon the medico-legal question of malpractice; and he is, therefore, peculiarly entitled to the thanks of both professions for having taken the lead in this interesting and important field of medical jurisprudence. Great Britain has certainly never furnished any work upon the subject, and the same remark is, we believe, strictly true in regard to the continental nations of Europe. One reason, perhaps, why America has preceded all other countries in relation to this matter, is the fact that suits for malpractice have been peculiarly frequent in the United States, particularly of late years. Commencing in Massachusetts forty years ago, with the celebrated case of Charles Lowell, of Lubec, concerning which Dr. John C. Warren published a small book, developing the facts and testimony elicited during the trial, these suits have gradually spread over the other New England States, and from thence over New York, Pennsylvania, and the greater portion of the West and South, raging, at times, like a kind of epidemic, every case decided in favor of the plaintiff serving as a fresh impulse to a new prosecution. Within the last few years the number of these suits has, if we mistake not, been very decidedly on the decrease, owing, mainly, to the circumstance that the prosecutor has generally been foiled in his attempt to extort money from his attendant for his alleged misconduct, or want of skill and attention. Were such suits confined to the miserable charlatans and pretenders of our profession, there would be just cause for their institution; but, unfortunately, it but too often happens that they involve men of the highest character for honesty and scientific attainment, men who would be an ornament to the profession in any part of the world. What is still worse, and a thousand times more humiliating, is that they are frequently, if indeed not generally, instigated by bad and designing practitioners, jealous of their neighbors and intent upon their ruin. We speak perfectly advisedly when we make this remark. We have never seen or heard of an instance of a suit for malpractice where this agency was not more or less prominent. On the other hand, we are equally certain that such prosecutions would rarely occur if lawyers always honestly discharged their duty to the medical profession; but, instead of this, they, like the jealous and unprincipled doctors, are too often ready and willing to foment the discontent of their clients and to divide the spoils, the result of an unrighteous suit, involving the defendant in great expense, in interminable trouble, and, not unfrequently also, in loss of character and of practice. We envy no lawyer his "contingent" fee in such a case, nor the comfort accruing to his tortured conscience when he comes to reflect, in his sober moments, upon his disgraceful conduct. It is high time that these unhallowed prosecutions, we should rather say persecutions, should cease; or, if they must be continued, that they should be conducted at least with a tolerable show of fairness and honesty, and not by men who are totally ignorant of the very rudiments of medical science, and who are, consequently, wholly incompetent to examine the medical witnesses that are always summoned upon such occasions.

The work of Dr. Elwell will do a great deal of good in pointing out the respective duties of the medical and legal professions in cases of suits for malpractice; and also in regard to the manner in which physicians and lawyers should deport themselves toward each other, when the former are subpœnaed as witnesses or experts in matters of ordinary medical jurisprudence. The chapters on medical evidence are very full upon this subject, and comprise, in fact, all that is really important to be known. We have nowhere, in the English language, seen the subject treated in so clear and satisfactory a manner within the same compass. The author's former experience as a practitioner of medicine and surgery, and his observation at the bar, have rendered him thoroughly conversant with the duties and requirements of medical witnesses, and the proper conduct of lawyers in trying their cases with a view to the attainment of the ends of justice.

We should be glad to follow the learned author in the discussion of the various topics of his novel and interesting treatise; but having lately noticed at much length the excellent work upon medico-legal medicine, by Briand and Chaudé, and, still more recently, the great work of the lamented Beck, edited by Dr. Gilman, of New York, we feel as if it would be needless to enter into details. The book is divided into two parts, the first being devoted to malpractice, and the second to medical evidence. Among the more important subjects discussed under the former head, are the general principles of law applicable to medical men; inherent difficulties of medicine and surgery; malpractice in amputations, fractures, dislocations, and ophthalmic medicine and surgery; the responsibilities of druggists; criminal practice; and abortion, or fœticide: under the latter division, Dr. Elwell speaks of the whole subject of medical evidence; of insanity in all its relations, including delusion and moral insanity; of mental incapacity invalidating a will; of poisons, especially arsenic and strychnine; infanticide; wounds; rape; coroner's office and inquests.

The above enumeration will afford a sufficient idea of the nature of the work under consideration. It should be added that it is furnished with a good index, and that it is illustrated with numerous adjudicated cases, both from American and English practice. It is well printed, and bound in law-book style, so that it exhibits the external characters rather of a legal than of a medical treatise; appearances also more in harmony with the author's present than past avocation.

In concluding this brief account of Mr. Elwell's book, we bespeak for it an extensive circulation and an attentive study, both on the part of the medical and legal professions, convinced that if they will only carry out its teachings, they will be wiser in knowledge, more courteous in manner, and more likely to subserve the great ends of justice during trials involving their mutual aid and co-operation. The work deserves a place in every medical and law library.

